# Bone Remodeling of Maxilla after Retraction of Incisors during Orthodontic Treatment with Extraction of Premolars Based on CBCT Study: A Systematic Review

**DOI:** 10.3390/jcm13051503

**Published:** 2024-03-05

**Authors:** Anna Ewa Kuc, Jacek Kotuła, Jakub Nawrocki, Maria Kulgawczyk, Beata Kawala, Joanna Lis, Michał Sarul

**Affiliations:** 1 Department of Dentofacial Orthopedics and Orthodontics, Wroclaw Medical University, Krakowska 26, 50-425 Wroclaw, Poland; j_kotula@poczta.onet.pl (J.K.); beata.kawala@umw.edu.pl (B.K.); joanna.lis@umed.wroc.pl (J.L.); 2 Dental Star Specialist Aesthetic Dentistry Center, 15-215 Białystok, Poland; kulgawczyk.m@gmail.com; 3 Department of Integrated Dentistry, Wroclaw Medical University, 50-425 Wroclaw, Poland; michal.sarul@umw.edu.pl

**Keywords:** bone remodeling, retraction, orthodontic treatment, CBCT study

## Abstract

**Background:** Incisor retraction is often a crucial phase in ongoing orthodontic treatment, with significant implications for alveolar remodeling mechanisms. There are two prevailing theories which seek to explain this. According to the first, teeth move with the bone, while according to the second, teeth move within the bone. This systematic review seeks to assess morphometric changes in the maxillary alveolar process resulting from incisor retraction following premolar extraction and to evaluate the potential for bone remodeling associated with orthodontic movement. **Methods:** The study was conducted following PRISMA (Preferred Reporting Items for Systematic Reviews and Meta-Analyses) guidelines. The following electronic databases were searched: PubMed, Google Scholar, Web of Science EMBASE and the Cochrane Central Register of Controlled Trials. The databases were searched using the following keywords: “Bone remodeling and retraction of incisors”, “Alveolar bone and incisor retraction”, “Bone thickness and incisor retraction”, and “Bone changes and orthodontic treatment”. Search filters were utilized to identify relevant papers and articles written in English and published during the last 10 years. Based on the information provided in their abstracts, papers and articles were selected according to the following criteria: randomized clinical trials (RCTs), controlled clinical prospective trials (CCTs), and retrospective studies. Articles unrelated to the study’s scope or failing to meet inclusion criteria were excluded. These generally comprised individual case reports, case series reports, literature reviews, experimental studies, studies with limited data (including conference abstracts and journal writings), studies involving an unrepresentative group of patients (less than 10 patients), studies concerning patients with syndromes, and animal experiments. The remaining articles which were deemed relevant underwent comprehensive reference review and such journals as the American Journal of Orthodontics, Dentofacial Orthopedics, International Orthodontics, Journal of Clinical Orthodontics, and Angle Orthodontist were manually searched. **Results:** Seven articles meeting the inclusion criteria articles were selected for final evaluation, with a total of 284 participants, including 233 women and 51 men. During the analysis of the results included in the publications, a lack of homogeneity was observed, rendering a reliable statistical analysis and heterogeneity assessment unobtainable. Noteworthy disparities in methodologies and measurements posed a risk of drawing inappropriate conclusions. Consequently, emphasis was placed on qualitative analysis, emphasizing the need for standardization in future studies of a similar nature, to enable valid and comparable analyses. **Conclusions:** The research findings incorporated in this review demonstrate that significant bone loss occurs because of incisor retraction, which diminishes distance between the bone surface and the root surface on the palatal aspect. The magnitude of this change may vary, contingent upon both the extent of incisor displacement and alterations in their inclination, thereby affecting the positioning of the root tips. This change is significantly higher in adults than in growing adolescents. The rationale behind this assertion lies in the widely recognized phenomenon of declining cellular activity with advancing age. The decrease in the speed and intensity of cellular changes may explain the diminished capacity for remodeling as patient age increases. There is ongoing discourse regarding alterations in the volume of bone on the labial aspect of the alveolar process. Further research is necessary to measure whether bone remodeling during orthodontic movement is contingent upon other factors, such as the speed and biomechanics of retraction, the level of applied orthodontic force, and the patient age.

## 1. Introduction

Orthodontic treatment encompasses not only the correction of malocclusion and enhancement of dental arch aesthetics, but also the preservation or restoration of optimal function and periodontal tissue health [1]. Smooth movement of teeth to their planned and stable position is contingent upon sufficient support of the alveolar process [2]. When planning tooth positioning, the anatomical limitations of the alveolar bone should be considered to avoid iatrogenic consequences in the form of dehiscence, fenestration of the alveolar process, or resorption of tooth roots [3].

There are many theories regarding bone remodeling during orthodontic tooth movement. Among them, two concepts are widely regarded as the most dependable: tooth movement “with the bone” and tooth movement “through the bone.” If, where the force is applied, resorption occurs in the pressure zone, and bone apposition occurs in the traction zone, then we are dealing with the movement of the tooth “with the bone”. The tooth then remains surrounded by the alveolar bone. In the case of tooth movement “through the bone”, we are dealing with an imbalance between bone resorption and apposition. In this situation, the tooth violates the bone boundary, remaining partially beyond its reach. The morphology of periodontal tissues, differences in bone density, inclination and position of teeth, and direction and magnitude of orthodontic force determine the type of bone reaction [4,5,6,7].

Retraction is a common procedure performed during orthodontic treatment, especially in the anterior maxilla [8]. Patients with a narrow alveolar bone width constitute a group that may show significant loss of periodontal tissue, especially alveolar bone, during incisor retraction [9]. The thickness of the alveolar bone around the incisors is an important factor in determining the direction of tooth movement. Morphometric assessment of the alveolar bone should be an indispensable element when planning orthodontic treatment, including changing the position of the incisors to avoid undesirable effects in the form of bone loss, dehiscence, fenestration, or root resorption [10]. Moreover, previous research has confirmed that in the group of orthodontically treated patients, the incidence of various types of bone dehiscence is higher than in the general population [3].

The use of cone beam computed tomography (CBCT) allows for a detailed assessment of the dimensions of the alveolar bone. Owing to their two-dimensional nature, commonly used cephalometric images exhibit significant limitations in periodontal tissue assessment [11,12]. Two-dimensional images are characterized by overlap, magnification and consequent distortion of anatomical structures, rendering accurate assessment of morphometric bone changes before and after orthodontic treatment unfeasible. The three-dimensional imaging method (CBCT) eliminates the problem of overlapping anatomical structures, allowing for detailed qualitative and quantitative verification of the alveolar bone and assessment of changes in the position of teeth. Imaging using CBCT can provide a detailed and reliable presentation of alveolar bone dimensions and is currently the best tool for planning orthodontic tooth movements [1,4,9]. Given the radiation risk and the imperative for patient radiological protection, careful consideration of the cost vs benefits associated with heightened tissue radiation absorption should underpin CBCT diagnostic planning.

The aim of this study was to present the results published in scientific publications assessing morphometric changes in the anterior part of the maxillary alveolar bone that occurred because of orthodontic retraction of incisors after premolar extraction, based on CBCT images. The null hypothesis was that tooth movement during retraction follows the “through the bone” pattern of movement.

## 2. Materials and Methods

### 2.1. Selection of Material

This systematic review has been registered in the PROSPERO database under the identification number CRD42023406039.

The study was conducted following the PRISMA (Preferred Reporting Items for Systematic Reviews and Meta-Analyses) guidelines. The study design was defined using the following PICO format:

Population (P): patients with full permanent dentition, encompassing both adolescents and adults;

Intervention (I): orthodontic extraction treatment with a fixed brace using incisor retraction;

Comparison (C): evaluation of the dimensions of the maxillary alveolar process before and after incisor retraction;

Outcome (O): identification of statistically significant/non-significant differences in the dimensions of the jaw alveolar process before and after treatment.

Electronic databases, including PubMed, EMBASE, and the Cochrane Central Register of Controlled Trials, were searched using the following keywords:

- Bone remodeling and retraction of incisors;
- Alveolar bone and incisor retraction;
- Bone thickness and incisor retraction;
- Bone changes and orthodontic treatment.

**Figure 1:**
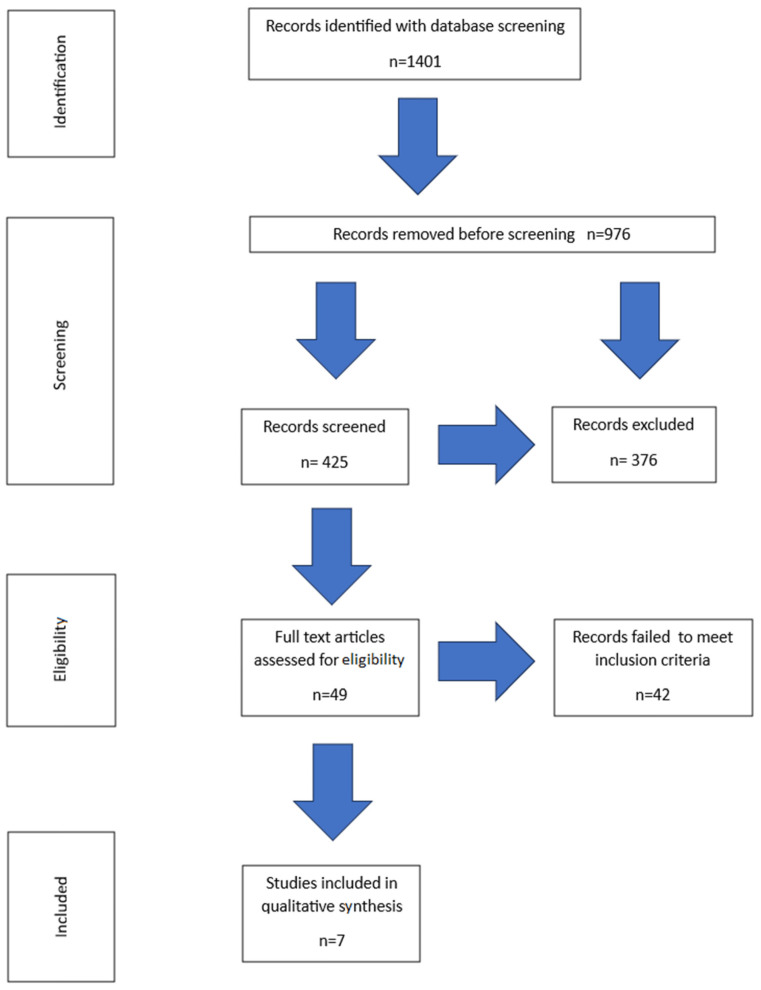
PRISMA flow chart.

Search filters were utilized to identify relevant papers and articles written in English and published during the last 10 years (before this date, only studies conducted on cephalometric images were detected). Independent searches of databases were conducted by the authors (A.E.K. and J.K.). Following the removal of duplicates, the titles were reviewed for relevance to the subject of this systematic review. Titles that passed this initial screening were then subjected to a detailed evaluation. During this review process, the authors were unaware of each other’s selections. Any discrepancies were discussed until an agreement was reached, with the involvement of the third author (MS) if needed. Based on the information provided in abstracts, papers and articles were selected according to the following criteria: randomized clinical trials (RCTs), controlled clinical prospective trials (CCTs), and retrospective studies. Articles unrelated to the study’s scope or failing to meet inclusion criteria were excluded. These generally comprised individual case reports, case series reports, literature reviews, experimental studies, studies with limited data (including conference abstracts and journal writings), studies involving an unrepresentative group of patients (less than 10 patients), studies concerning patients with syndromes, and animal experiments. The remaining articles which were deemed relevant, underwent comprehensive reference review and such journals as the American Journal of Orthodontics, Dentofacial Orthopedics, International Orthodontics, Journal of Clinical Orthodontics, and Angle Orthodontist were manually searched (Figure 1). The following data were extracted from reviewed articles: year of publication, group size, patient malocclusion, characteristics of treatment and control groups, the assessment method, and results.

### 2.2. Risk of Bias

The collected articles were subjected to risk of bias analysis according to Liu et al. [13], utilizing the ROBINS-I tool.

**Figure 2:**
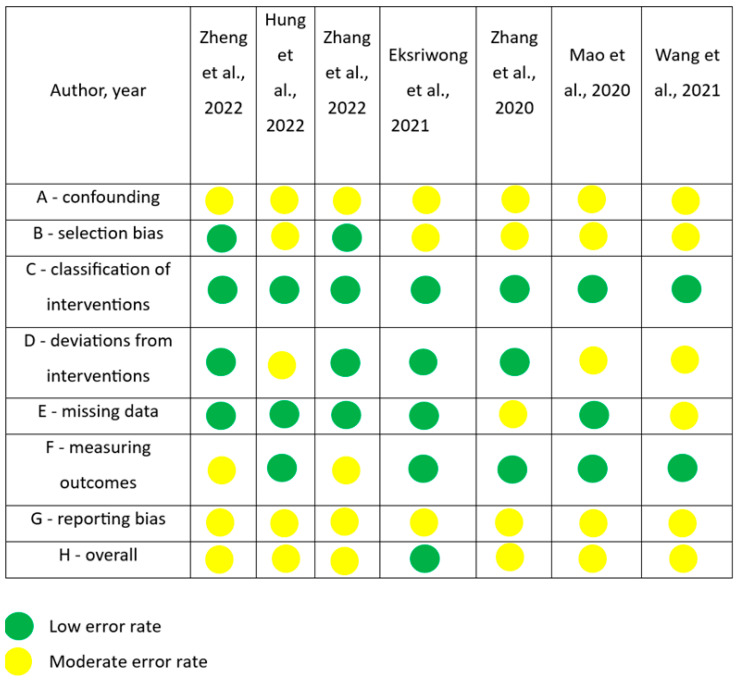
The risk of bias analysis of articles evaluating bone remodeling of maxilla after retraction of incisors during orthodontic treatment with extraction of premolars [14,15,16,17,18,19,20].

The quality and internal relevance (level of reliability) of each publication were rated as high, moderate, or low. Levels of evidence and criteria for evidence synthesis were as follows:

- High level of evidence;
  Studies were classified as having a high level of evidence if they met all the following criteria:
  ○ An independent blind comparison of the test and reference methods was performed (in Figure 2, marked as A).
  ○ The population was described in such a way that the disease status, prevalence, and severity of the disease were clear. The spectrum of patients was like the spectrum of patients in whom the research method would be used in clinical practice (marked as B in Figure 2).
  ○ The results of the test method had no impact on the decision to use the reference method (marked as C in Figure 2).
  ○ The test and reference methods are well described in technical and implementation terms (marked as D in Figure 2).
  ○ The assessments (observations and measurements) performed were well described, providing the diagnostic criteria used as well as information and instructions for observers (marked as E in Figure 2).
  ○ The repeatability of the test method is described for one observer (intra-observer performance) and several (minimum 3) observers (inter-observer performance) (marked as F in Figure 2).
  ○ The results are presented as relevant data needed for the necessary calculations (marked as G in Figure 2).
- Moderate level of evidence;
  Studies were rated as having a moderate level of evidence if any of the above criteria were not met. On the other hand, a study showing any of the deficiencies described below was rated as low evidence.
- Low level of evidence;
  Studies were considered to have a low level of evidence if they met any of the following criteria:
  ○ Assessment of the test and reference methods was independent (A).
  ○ The population has not been clearly described, and the spectrum of patients is disturbed (B).
  ○ The results of the test method influenced the decision to use the reference method(C).
  ○ The test, reference method, or both, were not well described (D).
  ○ The results were not well described (E).
  ○ The repeatability of the research method was not described or was described only for one observer (F).
  ○ The results may have a systematic bias (H).
  ○ The results were not presented in a way that would enable the calculation of effectiveness (G).

**Figure 3:**
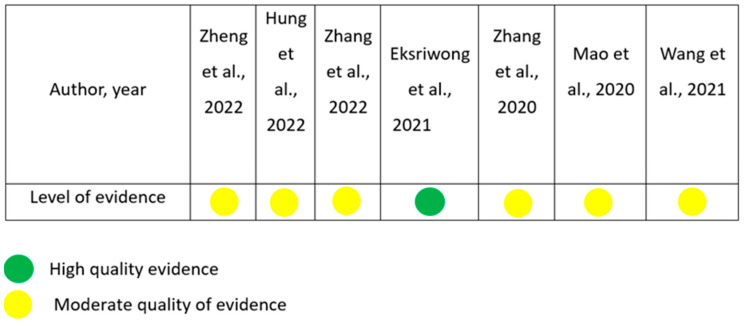
The evaluation of the conclusions according to the degree of evidence of articles discussing bone remodeling of the maxilla after retraction [14,15,16,17,18,19,20].

The evaluation of the conclusions according to the degree of evidence of articles discussing bone remodeling of maxilla after retraction was performed using the risk of bias table in RevMan 5.3 for RCTs (Figure 3).

### 2.3. Statistical Analysis

During the analysis of the collected data, a significant limitation was encountered impeding the execution of a dependable statistical analysis and the evaluation of heterogeneity. This impediment stemmed from the lack of coherence in results across individual studies. Differences in methodology, patient inclusion criteria, and methods of measuring and classifying outcomes were sufficiently defined to preclude any endeavor to amalgamate these data, thus risking fallacious deductions.

Without consistent and comparable data, the risk of distorting the results of statistical analysis increases significantly, which may consequently affect the credibility and scientific value of the results. Consequently, a qualitative analysis of available data was chosen as the basis for conclusions, with an emphasis on advocating for standardization in future research endeavors, in order to facilitate precise and reproducible statistical analysis.

## 3. Results

Keyword entries yielded 1401 abstracts. In total, 49 articles were initially confirmed as eligible for systematic review and were analyzed in detail. Out of the 49 full-text articles assessed for eligibility, 42 articles were rejected because the studies they contained did not relate to the method involving premolar extraction, or they solely relied on cephalometric analysis. Ultimately, seven articles were selected. The full selection process is shown in Figure 1.

### 3.1. Groups

#### 3.1.1. Group Size

The study group included 284 people, 233 women and 51 men. The average group size was 40 people. The largest group was found in Zhang’s article—72 people. The smallest group was included in Eksiwrong’s study—17 people (Table 1).

#### 3.1.2. Population

In the majority of studies, the participants were adult patients; however, in the studies by Zheng et al., Mao et al., and Wang et al. adolescents constituted the study cohort. In total, 284 individuals were enrolled for the study, comprising 233 women and 51 men (Table 1).

#### 3.1.3. Intervention

The inclusion criteria for patients in all of the analyzed studies were as follows: extraction of the first maxillary premolars, absence of significant medical history, no periodontal disease, no history of dental trauma, and the performance of CBCT before and after orthodontic treatment. Wang et al., Zheng et al., and Hung et al. also included patients with mild crowding in the arch in their studies. The treated patients underwent extraction of the maxillary first premolars to obtain space for the retraction of the incisors and canines. Skeletal anchorage was used in the studies by Hung et al., Mao et al., and Wang et al. Additionally, Mao et al. and Zheng Y. et al. used self-ligating brackets. Eksiwong et al. used a T-loop archwire for retraction purposes (Table 1).

### 3.2. Outcome

The main parameter was a change in the thickness of the maxillary alveolar bone on the vestibular and palatal sides.

In all studies, a correlation between incisor retraction and changes in alveolar process thickness was observed (Table 2).

As a result, in all studies, maxillary bone resorption occurred on the palatal side during incisor retraction. The greatest resorption was observed on the palatal side among a group of adults in Zheng’s study (−1.59) (Table 1). The results of statistically significant tests are presented below:

Zheng et al. demonstrated bone resorption on the palatal side both in the retraction group of adults (average −1.46) and adolescents (average −0.64); however, resorption was much greater in adults. Although bone thickness increased on the vestibular side in both groups, the increase was smaller in adults (mean +0.17) than in adolescents (mean 0.33) [14].

Hung et al. showed resorption on the palatal side (average −0.94) and an increase in bone thickness on the vestibular side (average 0.55); however, they did not observe changes in the total thickness of the alveolar bone before and after orthodontic treatment (average −0.27). Additionally, they noticed a significant bone reduction both vertically and horizontally on the palatal side [15].

**Figure 4:**
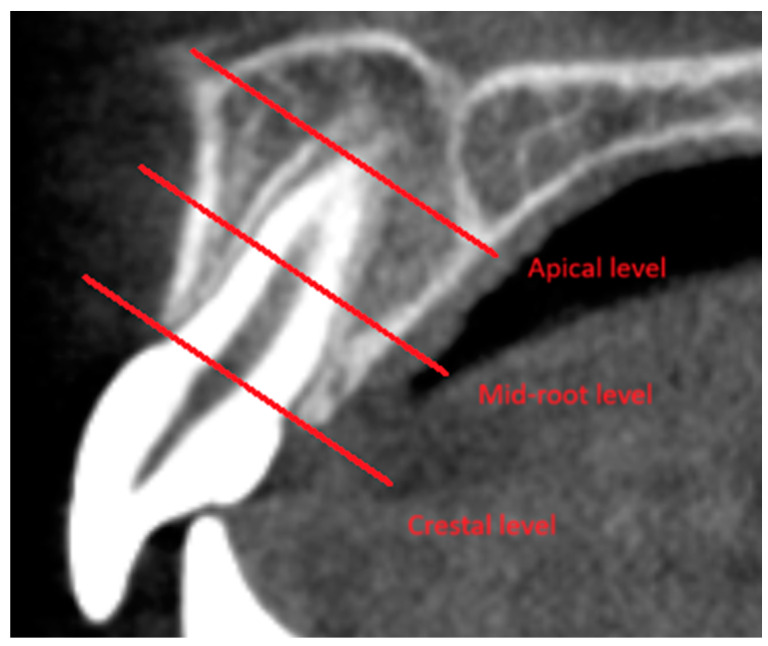
Three levels of measurements [14,16,17,19,20].

Zhang C. et al. showed alveolar bone resorption both on the vestibular side at all levels (average −0.35) and on the palatal side at the middle level (average −0.35). (Figure 4) They did not notice any difference in changes depending on gender, age, or duration of orthodontic treatment [16].

Eksiwong et al. noticed that on the vestibular side, the ratio of remodeling consisting of bone resorption to the amount of tooth movement is 1:1, while on the palatal side, it is 0.2−0.4. The bone on the palatal side does not seem to change, and only the distance between the root and the lamina compact palatine changes. However, the inclination of the incisor root is the only factor influencing the change in bone volume [17].

Zhang F. et al. demonstrated significant changes in the shape and thickness of the alveolar bone after incisor retraction. The process becomes thicker on the vestibular side, except at level 1. Bone resorption occurs on the palatal side (average −1.09) [18].

Mao et al. noticed that on the vestibular side, there is an increase in alveolar bone after retraction or it does not change (on average 0.1), with simultaneous bone resorption at all levels on the palatal side (on average −0.6) and a decrease in its height between the T0 and T1 levels. The greatest bone resorption was found at the crestal level on the palatal side (average −0.9) (Figure 4). They showed correlations between the displacement of the incisor root apex and bone resorption on the palatal side [19].

Wang et al. did not notice any changes in bone thickness on the vestibular side (average 0.1), but on the palatal side, they found a significant decrease in bone thickness (average −0.7). However, after a retention period of 18–24 months, bone reconstruction took place at the L1 level, with no changes at the other levels. (Figure 4) Additionally, they showed a significant reduction in the height of the process on both sides after orthodontic treatment, which persisted after the retention period [20].

## 4. Discussion

### Analysis of the Results

There has been a debate for many years about whether the biomechanics of orthodontic treatment cause the tooth to move in the bone or with the bone [21]. This is an important aspect when it comes to maintaining healthy periodontium and reducing the risk of incisor resorption in the case of extraction treatment of bimaxillary dentoalveolar protrusion, camouflage treatment in the case of class II malocclusions, or treatment of open bites. The use of maximum anchorage after premolar extraction and incisor retraction can significantly improve lip position, facial profile, and occlusion [22]. A large range of incisor displacement, unfortunately, comes with a high-risk of exceeding the so-called bone envelope, causing contact between the incisor roots and the palatal plate, the lamina compacta of the incisive canal, which may result in resorption of the incisor roots or fenestrations of the palatal plate [23,24]. Many factors, such as the amount of orthodontic force applied, the speed of tooth movement, the type of orthodontic movement—uncontrolled inclination, controlled inclination, or axial shift—as well as the patient’s age, influence bone remodeling [19]. The alveolar bone in young patients is very flexible during growth and quickly adapts to changes, while in adult patients, remodeling is significantly limited. This may be related to a reduction in the number of progenitor cells, reduced blood supply and density of fibroblasts, or a reduced ability of osteoblasts to proliferate and form bone [19]. Knowledge of the processes occurring during incisor retraction combined with the ability to precisely visualize the pre-treatment condition based on CBCT examinations allows for the development of an optimal orthodontic treatment plan for periodontal tissues and the bone envelope.

Undoubtedly, all studies analyzed in this review showed significant changes in the thickness of the maxillary alveolar bone after orthodontic treatment associated with incisor retraction. In all studies, a decrease in bone thickness was observed on the palatal side, while on the vestibular side, the results are varied and show both atrophy and gain. It has also been shown that the greater the distal root movement, the greater the changes in the volume of the alveolar process. Moreover, in children or adolescents, these changes are smaller, and bone resorption on the palatal and vestibular sides is not as severe as in adult patients. However, it was not observed that gender or the duration of orthodontic treatment had an impact on the changes occurring in the bone. Most studies are retrospective studies of medium evidentiary value. However, Eksiwrong’s research, as a prospective study, is characterized by high evidentiary value.

The research of Zheng et al. turned out to be very valuable and undoubtedly showed that age has a huge impact on changes in the volume of the alveolar process during retraction. Greater bone loss on both the vestibular and palatal sides was observed in adult patients than in adolescent patients. This suggests that orthodontic movement in adolescents may, according to the theory, take place with the bone, while after the end of growth in adults, the same movement will take place through the bone. This may explain the changes occurring during retraction in the alveolar process and guide treatment planning in adults, depending on the bone volume in the vestibular and lingual dimension. Moreover, the vestibular and palatal laminae can be treated as walls limiting the range of tooth movement. The results of these tests may also be an indication to start treatment as early as possible when bone remodeling capabilities allow for minimizing side effects or complications in the form of bone and incisor root resorption [14].

Research by Hung et al. showed no change in the total vestibulopalatal volume of the alveolar bone after incisor retraction. There was bone reduction on the palatal side, but this was accompanied by bone growth on the labial side. Such results indicating the lack of narrowing of the alveolar process after orthodontic treatment may be the result of the use of mini orthodontic implants for the axial retraction of the incisors. In these biomechanics, the incisors are not only retracted but also controlled in the vertical dimension, which may explain the lack of change in bone thickness at the appropriate levels. However, these studies confirmed the correlation between the extent of distal displacement of the incisors and bone thinning on the palatal side [15].

The research by Zhang C. et al., on the other hand, indicated that bone resorption occurs on both the labial and palatal sides as an end result of incisor retraction during extraction treatment. They did not observe bone regeneration on either side. However, the results may be subject to a risk of error since the measurements were made 2 weeks after the end of treatment. However, the processes of new bone formation take longer and are slower than bone resorption. Moreover, the study participants were exclusively adults. This may confirm that age has an impact on changes in alveolar process volume after orthodontic treatment and that the range corresponds to the amount of bone resorption [16].

Subsequent measurements performed by Eksriwong were of high evidentiary value, as they were prospective control studies, and they questioned the dependence of changes in the volume of the alveolar process on the extent of retraction and correlated it with the change in the inclination of the incisors. Contrary to previous studies by Cangialosi [25], the bone remodeling on the labial side occurred in a 1:1 ratio in response to tooth displacement, while on the palatal side, the bone did not change. Only the distance between the root and the palatal lamina compacta changed due to tooth movement. In these measurements, only the inclination of the incisors was a factor influencing the changes in the maxillary alveolar process. The advantage of the study was the use of skeletal structures as reference points for the measurements taken. The incisor axis can only be referenced if the change in inclination is less than 10°. In other cases, a change in inclination may falsify the measurement results [17]. The results obtained in this study align with Handelman’s research [26], which shows that the palatal plate of the maxillary alveolar process ought to be regarded as an orthodontic barrier not to be breached. The stability of the palatal cortical plate, regardless of the range of incisor movement, indicates that movement should be planned within the range of the process. Uncontrolled mechanics may predispose to fenestration, dehiscence of the alveolar process, and resorption of the incisor root tips as a result of greater retraction than the initial position of the palatal lamina compacta [17].

Interesting results were presented by Zhang F. et al. They showed that both on the labial and palatal sides, the alveolar bone drifts with the movement of the tooth, and the lingual bone crest moves apically. There is a significant reduction in bone volume on both sides, and on the lingual side, the loss reaches approximately one-third of the original bone height. Additionally, it has been observed that tooth tilt causes greater bone resorption than axial displacement. The extent of this tilt corresponds to the amount of resorption, but no direct correlation was found. This may be related to the fact that other factors, such as gingival phenotype or individual periodontal conditions, may influence the biological response resulting from orthodontic movement and make it impossible to find any mathematical correlation [18].

Mao et al. performed a retrospective cohort study, the results of which confirmed that because of orthodontic movement involving the retraction of the incisors, there is a significant reduction in bone thickness on the palatal side, and the displacement of the incisor tips is the main factor that determines the size of this change [19]. This confirms that while the alveolar process can undergo dynamic remodeling in growing patients, orthodontic movement in adults must be limited by the orthodontic walls of the cortical plates [27].

The most recent included studies as incorporated by Wang et al. confirmed significant atrophy of the palatine bone at all levels; however, no changes in the thickness of the process on the labial side were observed. These studies additionally showed that after almost two years of retention at the L1 level, there is an increase in bone volume on the lingual side, while no significant changes occur in other areas [20].

The above studies demonstrate that significant bone loss occurs on the palatal side because of incisor retraction. This amount may depend on both the extent of the incisor shift and the change in their inclination, and thus, the change in the position of the root tips. This change is significantly higher in adults than in growing adolescents. Cellular functions decline with age, which may explain the reduced ability to remodel as we age. Additionally, the rate of retraction may result in greater bone loss since repair processes may not keep up with resorption processes [20]. However, bone changes on the labial side are debatable. Further research is necessary, which would make the obtained measurements dependent on other factors, such as, perhaps, the speed and biomechanics of retraction, the applied orthodontic force, or the age of the patients.

The latest research conducted by Guo et al. may be optimistic, suggesting that despite the frequent occurrence of bone dehiscence on both the palatal and labial sides after orthodontic treatment using incisor retraction, the situation improves during retention by initiating bone remodeling. Additionally, spontaneous reorientation of the incisor roots was observed, which contributes to covering the fenestration and dehiscence with a thin layer of bone. In addition, the thick gingiva covering the palatal bone may enable bone tissue regeneration by preserving periodontal ligaments, which participate in bone remodeling. [28]

It should, therefore, be assumed that orthodontic movement in adults takes place through the bone, and most often, the bone does not adapt to the new position of the teeth. The palatal cortical lamina should be treated as an intact wall that limits the range of planned movement of the incisors. An additional limitation is the lamina compacta, which surrounds the incisive canal and may be the first to get in the way of the incisors during retraction and may also cause their resorption [23,24]. Advanced incisor protrusion should be treated as early as possible in the adolescent growth period when the body’s ability to remodel is high and when orthodontic movement occurs together with the bone. At this age, the incisive canal, the inclination of which is dependent on the inclination of the incisors, may also have a greater capacity for remodeling [23,24].

## 5. Conclusions

The studies show that because of incisor retraction, there is a statistically significant change in bone thickness. Significant bone loss is noted on the palatal side. This observed change may depend on both the extent of the incisor shift and the change in their inclination, and, thus, the change in the position of the root tips. This change is significantly higher in adults than in growing adolescents. Cellular functions decline with age, which may explain the reduced ability to remodel as we age. Additionally, the rate of retraction may result in greater bone loss since repair processes may not keep pace with resorption processes. The changes in the bones on the labial side are controversial, as they show both gains and losses. Further research is necessary to make the obtained measurements dependent on other factors, such as the speed and biomechanics of retraction, orthodontic force magnitude, and patient age.

## 6. Limitations

The primary constraints of this review encompass the inclusion of articles published exclusively in English within the last 10 years. This may affect the risk of statistical publication bias. Additionally, only studies that relied on 3D CBCT imaging were included.

## Figures and Tables

**Table 1:** Studies included in the systematic review.

Reference	Patients	Groups	Age	Patients Malocclusion	Treatment Method	Assessment Method	Results
Zheng Y. et al., 2022 [14]	N = 72 (F = 72)	G1 (minor) = 36 G2 (adult) = 36	G1 (minor) 11–16 years old G2 (adult) 18–35 years old	Bimaxillary protrusion with class I malocclusion	Extraction of four first Premolars, Self-ligating brackets	Pre- and post-treatment CBCT and late cephalograms	Changes of the alveolar bone thickness at the crestal, mid-root and apical third in the central incisor (adults): Labial: La3: 0.16 ± 0.44 La6: 0.09 ± 0.46 La9: 0.26 ± 0.85 Palatal: P3: −1.48 ± 0.79 P6: −1.70 ± 1.41 P9: −1.59 ± 2.67 Changes of the alveolar bone thickness at the crestal, mid-root and apical third in the central incisor (minors): Labial: La3: 0.38 ± 0.47 La6: 0.29 ± 0.42 La9: 0.32 ± 0.70 Palatal: Minors: P3: −0.88 ± 1.21 P6: −0.82 ± 1.82 P9: −0.33 ± 2.25
Hung et al., 2022 [15]	N = 24 (M = 6, F = 18)	G1 = 24	Mean ± SD 19.29 ± 4.64 years	Bimaxillary protrusion with skeletal Class I or II	Extraction of four first premolars, Incisor retraction treatment by sliding mechanics with microimplants in the maxilla	Pre- and post-treatment CBCT and late cephalograms	Changes of the alveolar bone thickness (ABT): Labial ABT: 0.55 ± 0.93 Palatal ABT: −0.94 ± 1.18
Zhang F. et al., 2020 [18]	N = 36 (M = 16, F = 20)	G1 = 36	Mean ± SD 20.6 ± 2.4 years 18–31 years old	Skeletal class I with bimaxillary protrusion	Extraction of four first premolars	Pre- and post-treatment CBCT and late cephalograms	Comparison of Alveolar Bone Thickness Before (T0) and After Orthodontic Treatment (T1): Labial side L1: T0: 0.70 ± 0.34 T1: 0.67 ± 0.58 L2: T0: 0.78 ± 0.30 T1: 0.94 ± 0.44 L3: T0: 0.88 ± 0.30 T1: 1.07 ± 0.54 L4: T0: 1.09 ± 0.46 T1: 1.50 ± 0.73 L5: T0: 1.87 ± 0.73 T1: 2.29 ± 1.06 Lingual side: L1: T0: 1.23 ± 0.58 T1: 0.51 ± 0.58 L2: T0: 2.03 ± 0.87 T1: 1.06 ± 0.96 L3: T0: 2.89 ± 1.14 T1: 1.79 ± 1.49 L4: T0: 4.09 ± 1.33 T1: 2.86 ± 1.92 L5: T0: 5.97 ± 1.70 T1: 4.55 ± 2.37
Eksriwong et al., 2021 [17]	N = 17 (F = 17)	G1 = 17	18 to 30 years old	on	Extraction of the maxillary first premolars, Incisor retraction was performed using T-loop	Pre- and post-treatment CBCT	Alveolar Bone changes (CT0 -CT1) at crestal, mid-root and apical Level of the maxillary Incisor Roots: Labial: Crestal: −1.3 ± 1.1 Mid-root: −0.9 ± 1.5 Apical: −0.8 ± 0.9 Palatal: Crestal: −0.2 ± 0.5 Mid-root: −0.1 ± 0.7 Apical: −0.2 ± 0.4
Zhang C. et al., 2022 [16]	N = 63 (M = 10, F = 53)	G1 = 63	Mean ± SD 24.41 ± 5.80 years 18–42 years old	on	Extraction of four first premolars	Pre- and post-treatment CBCT	The thickness changes in the maxillary alveolar bone at crestal, mid-root, and apical levels: Labial: A1: T0: 1.38 ± 0.76 T1: 1.13 ± 0.74 A2: T0: 1.71 ± 0.74 T1: 1.39 ± 0.77 A3: T0: 3.24 ± 1.36 T1: 2.75 ± 1.10 Palatal: B1: T0: 1.42 ± 0.69 T1: 1.41 ± 0.82 B2: T0: 2.91 ± 1.50 T1: 2.56 ± 1.58 B3: T0: 7.12 ± 1.76 T1: 6.74 ± 2.13
Mao et al., 2020 [19]	N = 38 (M = 7, F = 31)	G1 = 38	15–33 years old Mean age 19.52 years	Bimaxillary protrusion with class I malocclusion	Interactive self-ligating brackets, retraction using TADs	Pre- and post-treatment CBCT	The thickness changes in the maxillary alveolar bone at the crestal, mid-root, and apical third: Labial: Crestal: T0: 0.8 ± 0.3 T1: 0.8 ± 0.3 Mid-root: T0: 0.7 ± 0.2 T1: 0.9 ± 0.4 Apical: T0: 0.9 ± 0.3 T1: 1.0 ± 0.6 Palatal: Crestal: T0: 1.6 ± 0.4 T1: 0.7 ± 0.9 Mid-root: T0: 2.9 ± 0.8 T1: 2.2 ± 1.4 Apical: T0: 4.4 ± 1.4 T1: 4.2 ± 1.8
Wang et al., 2021 [20]	N = 34 (M = 12, F = 22)	G1 = 34	Mean ± SD 14.29 ± 1.24 years	Bimaxillary protrusion	Extractions of the four first premolars, miniscrews for maximum anchorage	Pre- and post-treatment CBCT	Comparison of mean labial and lingual alveolar bone thickness at T1 (pre-treatment), T2 (post-treatment) and T3 (retention phase) of the central incisor: Labial: Cervical level: T1: 1.53 ± 0.32 T2: 1.63 ± 0.57 T3: 1.61 ± 0.59 Middle level: T1 1.84 ± 0.50 T2: 1.95 ± 1.02 T3: 1.74 ± 0.52 Apical level: T1: 4.06 ± 1.35 T2: 4.07 ± 1.94 T3: 3.78 ± 1.31 Palatal: Cervical level: T1: 2.66 ± 0.77 T2: 1.88 ± 1.01 T3: 2.15 ± 0.60 Middle level: T1: 4.49 ± 1.58 T2: 3.75 ± 1.88 T3: 3.86 ± 1.34 Apical level: T1: 7.93 ± 1.87 T2: 7.33 ± 2.21 T3: 7.14 ± 1.74

**Table 2:** Bone remodeling after intervention.

	Zheng Y. et al., 2022 [14]	Hung et al., 2022 [15]	Zhang C. et al., 2022 [16]	Eksriwong et al., 2021 [17]	Zhang F. et al., 2020 [18]	Mao et al., 2020 [19]	Wang et al., 2021 [20]
Labial resorption					x		
Palatal resorption	x	x	x	x	x	x	X
Apposition/no labial resorption	x	x	x	x		x	X
Apposition/no palatal resorption				x			

## Data Availability

All relevant data are within the paper.
